# Culturally Appropriate Peer-Led Behavior Support Program for African Americans With Type 2 Diabetes

**DOI:** 10.3389/fpubh.2018.00340

**Published:** 2018-11-23

**Authors:** Florence O. Okoro, Shelby Veri, Valencia Davis

**Affiliations:** ^1^School of Nursing, University of North Carolina at Charlotte, Charlotte, NC, United States; ^2^Department of Public Health, University of North Carolina at Charlotte, Charlotte, NC, United States; ^3^Community Care Partners of Greater Mecklenburg, Charlotte, NC, United States

**Keywords:** peer support, peer supporter, support recipient, self-management, type 2 diabetes, culture, qualitative descriptive

## Abstract

Current literature poorly defines the specific ways trained peer supporter influences health care behaviors. This study attempts to identify the key defining features of a culturally appropriate peer support program for African Americans with type 2 diabetes by exploring participants experiences related to assistance with daily disease management, emotional support, linkage to clinic care and community resources, and ongoing support. We used a qualitative interpretive descriptive approach to collect data through semi-structured interviews from 20 African Americans with type 2 diabetes participating in a peer support program. Interviews captured participants' background and experiences with the peer supporter and evaluated the cultural appropriateness of the peer support intervention. Data was coded deductively using predetermined codes found in the peer support literature and inductively to identify emergent themes. Three specific themes were identified namely [1] healthy behaviors [2] frequent telephonic contact and [3] emotional support as a by-product of other support activities. These findings mirror the broader literature on what constitutes culturally appropriate peer support programs for ethnic minorities. We recommend the inclusion of culturally appropriate peer support program to complement diabetes management as targeted plan for improvement in clinical care and ultimately, diabetes outcome.

## Introduction

Diabetes affects over 29 million Americans (9.3% of the population) and this figure is projected to increase to 35 million in 2040 (1). The estimated economic cost of diabetes continues to increase from $245 billion dollars in 2012 (2), to $320 billion dollars reported in 2015 (1). Approximately, 13.2% of all Blacks bear a disproportionate burden of the disease (3). Compared to non-Hispanic Whites, African Americans are more likely to suffer from the complications of diabetes such as cardiovascular disease, kidney failure, and amputation and have increased mortality rates (4). African Americans also suffer a widening gap in health disparities and are reported to experience more barriers to diabetes self-management than non-Hispanic Whites (5). In addition, African Americans of low socioeconomic status experience the stress of financial insecurity, lack of quality health care, and are less likely to receive culturally relevant health education thereby limiting their understanding of disease, and reducing their potential to make healthy lifestyle decisions to effectively manage disease (6).

Peer support is broadly defined as social support provided by “someone like me,” who belongs to the same age group, disease state, or social group etc. In type 2 diabetes, peers are either people who have the disease or a caregiver of those affected by it—such as a spouse, parent or child of the person with diabetes. Peers affected by Type 2 diabetes (T2DM) can help support others affected by the disease with coping effectively with a range of demands and challenges involved in diabetes self-management. Organized peer support programs often recruit and train peers from the target community to enhance their ability to function as culturally sensitive peer supporters (7). The training covers a variety of skills such as problem solving, clear communication, decision making, identifying and accessing healthcare resources, comprehensive diabetes management principles, and the psychological impact of living with the condition. Wientjens (8) listed the core competences of peer supporters to include the ability to communicate clearly, a willingness to learn, confidence, flexibility and reliability.

Peer support can be an efficient and cost-effective means of sustaining a lifetime of self-management in patients with chronic diseases, such as T2DM (9, 10). Peer-delivered self-management support has been used to reach low-income groups and the minority populations who suffer health disparities and bear the highest burden of T2DM (3, 11, 12). Peer support interventions have demonstrated improvements in health behavior and significant reduction in hemoglobin A1C (HbA1C) as a measure of blood glucose control (9, 13).

Different cultural contexts influence health behaviors like diet, how we feel about diseases and health and how we give and receive support from others. Peers for Progress (www.peersforprogress.org) identified four functions to guide peer support activities and to standardize peer support around the world and proposes that peer support activities be tailored such that they are culturally appropriate to the participants and the setting (7). The four core functions that guide the provision of peer support services include:

Assistance in Daily Disease Management where peer supporters use their own experiences in helping people figure out how to manage their disease in the context of their daily lives.
Social and Emotional Support where peer supporters provide peers with opportunities to discuss personal problems, are empathetic, good listeners and offer advice without being judgmental. The peer supporter must promote use of skills by just “being there” and provide encouragement to stay motivated to reach self-care goals and also share experiences of what worked for them when they faced similar difficult and challenging situations in their lives.
Linkage to Clinic Care and Community Resources where peer supporters function to assist peers navigate the complex healthcare systems, through virtual (telephonic, texting, email) and face-to-face visits that are geared toward helping persons living with chronic conditions are linked to clinical care, and access to community resources.
Ongoing Support by providing long-term, flexible and consistent follow-up contact to keep the peers engaged in their self-care that encourages the individual with chronic disease to continue to maintain self-care, avoid disease complications, and live good quality.

The WHO 2008 report on “Peer Support Programs in Diabetes” concluded that there is still much to learn about how to optimally organize and deliver culturally appropriate peer support interventions, which types of programs are best for different populations in different geo-cultural settings. Since African Americans suffer disproportionately from the morbidity and mortality resulting from poorly managed T2DM, we employed an interpretive descriptive approach to elucidate the experience of African Americans in a culturally appropriate peer support program to enhance diabetes self-management with the aim of reducing emergency department visits, and diabetes related complications. The central research aim was to define the key features of a successful culturally appropriate peer support program for African Americans with type 2 diabetes.

## Methods

Qualitative descriptive design 14, 15, with purposeful sampling method was used to conduct an in-depth study of a culturally appropriate peer support program similar to one described by Neergaard et al. (16). We conducted in-depth key informant interviews and analyzed the data thematically.

Interviews were semi-structured to cover specific relevant topics in peer support and allowed flexibility in what and how probe questions were asked to ensure that interviewees' perspectives were fully captured as recommended by Creswell and Poth (17) and Sandelowski (15). This method proved useful in elucidating emergent themes through conversation and discourse, the purpose of which was to generate rich data regarding the experiences of support recipients in a peer-led self-management support program (15, 18).

The interview guide was a set of six questions with nested probe questions developed by the Primary Investigator who is very familiar with the peer support literature. The PI had prior proven success collecting rich data with interview guide developed from peer support literature. This instrument asked participants to describe their experiences in the peer support program. Specifically about their experience, receiving assistance to manage their disease daily, emotional support received, assistance with linking them to clinics and community resources, and ongoing long-term support to help them stay focused in their self-management. We asked participants to describe their engagement and response to peer support activities and to share any influence their culture or environment had in the perception of type of support they received or they will prefer to receive. Sample of interview question and prompts are included below;

Let us start by reviewing the support provided to you by the peer supporter

How will you describe the support provided to you by your supporter?

What makes you feel well supported?

### Participants and program

The Institutional Review Board (IRB) of the University of North Carolina at Charlotte approved this study. All participants who met study eligibility criteria provided signed informed consent to participate. We used purposive sampling to recruit participants from an existing peer support program that served African Americans with T2DM in an urban area who were mostly covered by public insurance (Medicaid). The aim of purposeful sampling was to select information rich cases for the study. Information rich cases were the participants that gave the required information, which helped to achieve the objectives of the study (17). Inclusion criteria were: participants had been diagnosed as having T2DM, were enrolled in an existing peer support program for at least 3 months, had attended monthly group meetings, received phone calls from the peer supporter, had the ability to communicate, and were willing to participate in the study. Participating in the peer support program for at least 3 months allowed sampling of participants who had been in the program long enough to be able to recount in-depth experiences.

Participants were patients from physicians' practices, outpatient clinics, and hospitals of a major healthcare organization in the region. They were typically referred to the peer support program by their health care team if they were newly diagnosed T2DM, had frequent visits to the Emergency Departments, self-report of struggling with maintaining their glycated hemoglobin (A1C) level below 7.0 mg/dl and/or had a BMI over 30. Participants were paired with a Peer Support Specialist who facilitated the monthly group meetings and called the support recipients at least twice a month. The Peer Support Specialist was usually a member of the participants target community, in this case African American and had T2DM. To qualify as a peer support specialist, you had to demonstrate ability to manage your disease well, a willingness to share your experiences, and to receive the appropriate peer support training.

### Data collection

Two trained research assistants conducted semi-structured individual interviews (17) between March 2016 and June 2017. We developed the interview guide based on the peer support literature and to meet the objectives of the study. After the first three interviews, the interview guide was revised in an iterative process. We conducted 19 interviews in the participants' homes and one in a fast food restaurant based on participants' preference. Each interview lasted between 30 and 45 min. We added participants in the study until thematic saturation was achieved and no new themes were identified. Participants were compensated with a $25 Walmart gift card for their participation in the study. All interviews were conducted face to face, audiotaped and transcribed verbatim. The PI and the trained research assistant validated the transcripts by re-listening to the tapes.

### Data analysis

After reviewing each transcript for accuracy, all transcripts were imported into *INVIVO* 10 software for coding and analysis. Data was deductively coded using predetermined codes found in the literature on peer support and inductively to identify emergent themes. We used thematic analysis process by iteratively reading and re-reading the data to identify inductive codes and to formulate themes. Two research team members separately reviewed the coding and we held weekly meetings to discuss and resolve any discrepancies. Data collection continued with the addition of new participants until we could not identify any new codes and therefore we achieved thematic saturation.

### Trustworthiness

We presented the emergent themes to seven of the research participants during one of their monthly meetings. There was consensus agreement on the themes. Trustworthiness of data Creswell and Poth (17), recommended member checking as a credible means of affirming trustworthiness of the data.

## Results

### Participants' demographics

Participants comprised of 13 Females, 7 Males. All participants were African Americans. Age ranged from 30 to 82 years; median age was 61 years. Six were married, 10 divorced, and 4 single. Education attainment of participants revealed 1 college graduate, 6 with some college, 9 high school graduate, 1 GED and 3 with some high school. Employment status—12 were unemployed, on disability, 4 were employed and 4 were retired.

### Themes

Three themes emerged from the data analysis: (1) Healthy behaviors (2) frequent regular telephone contact (3) emotional support—as a “by product” of the support activities rather than a separate category.

#### Theme 1: healthy behaviors

Assistance with daily disease management was primarily focused on advice on healthy behaviors. The healthy behaviors include healthy cooking, healthy eating right, and reading food labels followed by physical exercise. Examples of quotes from the participants were included to support each point that was discussed.

On cooking and eating, participants described how they were advised to mostly bake their foods and stay away from frying. Participants understood that frying their food items was unhealthy, boiling and baking were better and healthier. Switching from frying foods to baking helps to lower the consumption of fatty foods, which is recommended for patients with diabetes. Sample quote from a participant to support the advice to avoid fried foods is as follows;

“*Eating and cooking, she tells you not to eat as much grease and bake mostly, or boil it.”* (Participant 6).

Participants reported that they were advised on portion sizing. Portion is how much food we choose to eat at one time and it is totally under our control. Serving is the amount of food listed on a product's nutrition label. The peer supporter advised the participants that when they cook, they should eat a serving and save the left over for another time as this participant said and I quote “*She talks about portion sizes that kind of assist you when you're cooking and all that. Let her walk in here and my plate be running over. But it's not ever running over. Potatoes, rice, I backed out.”* (Participant 5).

The participants described how an emphasis was placed on their reading food labels when they go grocery shopping and how that will help them to know the nutritional content of what they were buying especially the sugar, fat, and cholesterol content. Food labels also help to know the nutritional content of one food item against another, facilitating comparison and aiding in making healthy choices. The peer supporter advised the participants to read all food labels before purchasing, to help them in cooking and portion sizing as reported by a participant with this quote “*Reading my foods and reading the labels. In fact, the last group meeting we were talking about when you are choosing certain foods, read the back of that label. Tell you what the contents is and how much, what you're eating and so forth and so, I've been doing that pretty regularly now.”* (Participant 4).

A participant stated that the peer supporters not only teach healthy eating and exercise but follows through to ensure compliance by observing them put what they learnt in practice by this saying “*Some of them will tell you what to eat and watch how you eat and stuff and then I go and do my exercises and stuff.”* (Participant 9). This account suggests that the participants were also visited in their homes.

The participants reported how they were encouraged to engage in physical activity within recommended limits depending on individuals' physical situations. Example is a part who just had surgery for hip replacement. The peer supporter calls up on phone to encourage her to exercise within her functional limitations. The patient said the following “*But you know I just come out of a hip replacement from a car accident many years ago so I'm just now beginning wanting to walk. At one time, I could not do it but I could sit at home, lift up my legs, and do that kind of exercise. And she might call and say, do you feel like a walk? I might say no. But she pushes me. If it's just out my front door to the parking lot because I couldn't do it. Because I've come a long, long way. A long way.”* (Participant 6).

Physical activity is an important component of the treatment plan for patients with T2DM. Physical exercise helps to control diabetes by keeping blood sugar in the correct range and in weight management. Participants reported that they were advised to keep active and exercise regularly as much as they can because of its health benefits, which includes weight loss, as this participant reported. “*Well I try to do what she asks me to do like when we were talking about my weight and stuff and me needing to lose weight because that would help my numbers come down too. She would call me and we would talk about it and it was kind of hard at first but then I started walking and stuff so I try to do whatever she suggests. If she suggests me to walk three times a week, that's what I try to do and I did start walking three times a week and I try to treat her like she treats me.”* (Participant 14).

#### Theme two: frequent regular phone call

Participants described how the peer supporter used phone calls to reach out to them for different purposes and to fulfill different peer support functions. Phone calls were used in addition to the face-to- face meetings to render most of the peer support services. There is the regular biweekly calls to all support recipients to find out how they are doing generally and to find out if they have problems with their diabetes self-care. One of the calls is also to remind them about the monthly group meeting and to encourage them to attend. Phone calls that are more frequent are made to individuals who need follow up on issues and those who have A1C's that are consistently high. Participants described how they felt good about the phone calls, which makes them have a sense of belonging that someone cares for them. The following quote support what the participants said about the frequent regular calls. “*Excellent, Ms. … calls me all the time and sometimes she calls me twice a week just to check on me and see how I'm doing and if I need anything or if there's anything she can do for me. She really supports us.”* (Participant 5).

“*They always concerned about different things like your sugar, your HbA1C, are you keeping up with those type of issues and that helps because I never been in that space where people call me to see how I'm doing and am I doing the correct things and its pleasurable to get that kind of attention.”* (Participant 12).

In the provision of emotional support, participants described how phone calls were one of the means to reach out to them, which they described as very good. Emotional support encourages compliance and helps patients with diabetes get through their daily lives. “*It's good to have someone to talk to when I'm home all the time by myself. When she calls, it just brightens my day up. She's just one of those people that when you hear their voice, ‘Hello Ms.……’ it just brightens up my day.”* (Participant 11).

The Peer supporter also called to remind the support recipients about the monthly meeting. One participant reported how she liked being called to remind her of the date/day of the monthly group meeting because she looks forward to attending the meeting. “*My favorite would be her calling me and telling me that we meeting this week. That would be my favorite because that I like. Like I said, I'm a go-getter and she'll call me and let me know the next one. I like her really being there.”* (Participant 4).

Participants were also encouraged to call the peer supporter if they needed. Participants reported that the peer supporter shared her private number and that made them feel comfortable to call her anytime. “*I can call her any day that I need something, she might not answer immediately but calling back in ten minutes”*. (Participant 14) The function of ongoing support was accomplished largely through phone calls, which could be weekly, biweekly, or monthly. The number of calls depended on who and why the call was made. One participant summarized the phone calls as “*She great on phone calls. I go, ‘Oh, Lord’ she great on them phone calls, yes, she do call, I can just look at the number and tell that's her, she's great”*. (Participant 2).

#### Theme three: emotional support was a “by product” of other activities

The participants' description of the emotional support they received was diverse and depended on their perception of the support and how it was delivered. In order words, emotional support transcended all the other types of support, making it difficult to give it a distinct classification. When asked to describe the emotional support they received from the peer supporter, the participants described what they perceived that made them feel emotionally supported. Participants described the peer supporter activities categorized under assistance with daily disease management, linkage to clinic care and community resources, and ongoing support as items that made them feel emotionally supported. Some quotes from the participants' responses are:

“*She offers me a lot of advice as far as when I first started with Ms. … I was sneaking eating and she would know it. One time she caught me. She did not get all aggressive or ugly like that. She just broke it down to me and brought it down to how important my health was and got me back on the right track.”* (Participant 13). This quote will be an example of providing assistance with daily disease management but the participant categorized it as emotional support.

“*Ms.…… always makes sure before you leave class, do you have any questions concerns and that helps a lot and I just think that I just end with this group.”* (Participant 14). The participant used this quote to describe what made him feel emotionally supported.

“*I think when I first met Ms. …, I was going through some mental stuff and she talked to me, talked me down and got me started going to see somebody about the problems that I was having so it's working out really well because I do feel better.”* (Participant 10). This participant described how the peer supporter linked her up with the resources that helped her to solve her personal problems. According to the participant, this him/her feel emotionally supported.

“*Excellent, Ms. … Calls me all the time and sometimes she calls me twice a week just to check on me and see how I'm doing and if I need anything or if there's anything she can do for me. She really supports us.”* (Participant 2). To this participant, calling regularly, like twice a week made her feel emotionally supported.

One participant described how listening and cutting down on his food helped him not to go crazy and as such gave him psychological support.

Participants were asked follow-up questions based on their responses to the main questions. Some of the probe questions were: *Which is your favorite type of support and why? Which type of support do you value the most and why? Which type of support do you enjoy receiving and why?* Most participants who chose emotional support as answer to the questions described emotional support in many ways. Examples of some of the probe questions and the responses were:

### Which is your favorite type of support and why?

Most of the participants chose emotional support as their favorite type of support. Three quotes below from the participants are examples of how they described emotional support as their favorite type of support;

“*My favorite support is her being there just to talk to me like if I run into a problem or if I don't understand, I can call her and she'll stop what she's doing and just go over it over the phone with me and that's my favorite support when she's there for me.”* (Participant 12).

“*Well, because she's there for me and I like that because I can call her and say, I'm having trouble like my glucose is not saying what it's supposed to say. And she would say, Well check it again after you eat or check it before you eat this time. I can't think of anything, she's always there. At the end of the day I see some light at the end of that tunnel.”* (Participant 12).

“*Well my favorite would be her calling me and telling me that we meeting this week. That would be my favorite because that I like. Like I said, I am a go-getter and she will call me and let me know the next one. I like her really being there.”*

However, one participant stated that his/her favorite type of support is ongoing support, which he/she described as “being there.” Generally, the phrase “being there for me” is regarded as emotional support. “*My favorite is ongoing support, to know that she's going to be there still.”* (Participant 14).

Most participants did not respond to the following three probe questions because the stated that it is repetitive as they looked like the questions asked previously and they do not have a different response. The interesting finding is that the four participants who responded to the questions all said that emotional support was the support they most valued, support they enjoyed receiving the most, and the support they will like to be provided with most of the time. The quotes that the participants used to describe the emotional support are listed below as follows:

### Support most valued and why?

“*Emotional because your day is up and down. Some days you feel good and some days you do not feel so good so it is always good to have someone to call when you are not feeling so good. Even when you are good, you just want to touch base because they are always there so you can always call. It's a lot to worry about with your sugar up and down and you have to take control over it”*. (Participant 17). In peer support literature, this quote will be an example of providing ongoing support but the peer support recipient described it as emotional support.

“*Emotional and clinical and just being supportive. Her calling me and sometimes I sit at home because I am disabled, I be there by myself and I just be needing somebody to call and it seems like she knows and its Ms. … and it really helps a lot. The diabetes classes, they help a lot.”* (Participant 10). In peer support literature, regular phone calls to the patients is categorized as providing ongoing support, diabetes education classes is categorized as informational support. It is interesting to note that the patient describes these as giving him/her emotional support.

### Support enjoy receiving the most and why?

“*Because a lot of times I've been unhappy with myself for a long time. I was not pleased, it was not so much of me just being overweight, but it is just that when I would get depressed she was just there for me, and that to me is number one. To bring light back into my life and just let me know that this can change.”* (Participant 6). This quote from a participant is a typical example of what is categorized as emotional support in the peer support literature.

### Support you will like provided with the most and why?

“*Emotional because it's good to have someone to talk to when I'm home all the time by myself. When Ms.…calls, it just brightens my day up. She's just one of those people that when you hear their voice, ‘Hello Ms….’ it just brightens up my day.”* (Participant 20). In peer support literature, this quote from a participant will be categorized as both emotional and ongoing support but this participant identified it as what made him/her feel emotionally supported.

Emotional support emerged from the way the support was rendered suggesting that this form of support may not be categorized separately but rather a description of the mechanism of support exchange. We therefore, divided emotional support into sub-themes to highlight the mechanisms as shown in Supplementary Table 1.

## Discussion

We used a qualitative descriptive approach to explore the experiences of African Americans in a peer support program for people with T2DM. Three themes emerged from the data analysis. These include, healthy behaviors, frequent and regular phone calls, and emotional support became apparent from how the support services were provided rather than a separate category.

The themes that emerged from the data analysis were from the descriptions of the functions of peer support as perceived by a population of African Americans with T2DM who attended a culturally appropriate peer support program. The program was designed to be culturally appropriate because the peer support specialist was from the target community, understood the culture of the people, was of equal standing, had the same illness condition and was able to connect with the group in a way acceptable to them. Assistance with daily disease management focused on information on healthy cooking, healthy eating, and physical activity. The knowledge translation was tailored to the specific needs of the target population, taking into consideration their cultural beliefs and language requirement. The peer supporter exchanged knowledge that emerged from her experiences of living with the illness, the type of knowledge that healthcare providers maybe ignorant of because it came from real life experiences (19).

The peer support program was acceptatble to the participants because it was culturally appropriate in terms of use of language, advice on cultural diet, social emphsis, and incorporation of cultural health beliefs. This finding aligns with the literature on culturally appropriate peer support programs for minority ethnic population. Brown et al. (20) reported that culturally appropriate diabetes self-management education for Mexican Americans was acceptatble and showed significant improvement in diabtetes knowledge.

In a systematic review, Attridge et al. (21) found that culturally appropriate diabetes education for ethnic minority with type 2 diabetes led to improvements in glycemic control, knowledge, and diabetes self-efficacy. This is demonstrates evidence that medical intervention complemented with culturally appropriate peer support program in diabetes management provided the long-term ongoing support necessary to sustain improved diabetes outcomes (7, 13, 22).

In addition, African American women with type 2 diabetes in a focus group discussion on eating habits reported that they eat regularly, unable to apply self-control to eating and only sense of fullness compel them to stop eating (23). In related studies, African American women despite being more likely to express willingness to adhere to recommended dietary regimen than other groups, continue the consumption of foods high in energy, saturated fats, and sugars (24–26). It appears African Americans especially women struggle with eating healthy and this may be the reason the peer supporter laid emphasis on cooking and eating healthy in the provision of the function of assistance with daily disease management.

People with diabetes often experience emotional distress especially when their blood glucose reading remain suboptimal regardless of their perceived efforts to adhere to recommended regimen (27). Therefore, people with diabetes need non-judgmental support to help them cope with lifestyle modifications and fear of uncertain future. These interviews with African American adults who participated in a culturally appropriate peer led diabetes self-management support program revealed findings consistent with studies that reported that emotional support resulted from how the support was delivered rather than a distinct category (28, 29). The participants described the encounters that made them feel emotionally supported. Activities described were calling them to remind them of the date for group meeting, listening and advising, linking them to resources they need for the daily disease management, encouraging them to do physical exercise, ongoing support through phone calls and just being there. The listed activities overwhelmingly fulfill the other three categories of functions of peer support identified by Peers for Progress, suggesting that emotional support is a by-product of peer support activities.

### Implications for practice

Culturally appropriate Peer delivered self-management support for African Americans with type 2 diabetes seems promising for better diabetes self-management outcomes. In addition, culturally appropriate peer support program complementing diabetes medical management may be a means of reaching the hardly reached like low-income African Americans and thus help in closing the health disparity gap that affect this population. We suggest the inclusion of peer support program to complement diabetes management as targeted plan for improvement in clinical care and ultimately, diabetes outcome.

### Limitations of the study

The purposive sample may not be a true representation of the population of adult African Americans with type 2 diabetes. The purposeful sampling technique utilized for the study was to recruit typical samples of those who will provide rich information about the peer support they received. The data collected maybe biased due to sampling error. This study will only be generalized to those that share the same characteristics as the sampled participants. However, the aim of the sampling was to get information-rich cases that provided comprehensive understanding of the phenomenon that we studied.

We conducted the interviews in participants' homes, restaurants, and community settings. The setting of the study may have influenced the responses of the participants. Member checking was done with seven of the participants who unanimously agreed on the themes that emerged from the study.

Another limitation of the study is that only 20 participants were interviewed. The participants were sampled from one peer support program, which adds to the strength of the study because the sample of participants is specific to the program and they can speak to its effectiveness. In addition, data was collected until information power was achieved.

In conclusion, despite the stated limitations, this qualitative descriptive study clearly demonstrated that culturally appropriate peer support program might be beneficial in improving type 2 diabetes outcomes in the African American community. Also culturally appropriate peer support program maybe an excellent means to reach low-income groups and minority populations like African Americans who suffer health disparities and bear a high burden of T2DM (11, 12).

## Author contributions

FO was the PI for the research project; contributed to the the design, data collection, analysis, and results; wrote the report, and contributed to the result and discussion sections of the manuscript, as well as editing the entire manuscript. SV contributed to the collection of data, analysis, and the writing of the report; wrote the literature review of the manuscript and edited the manuscript. VD contributed to the design and data collection; wrote the methods section of the manuscript and edited the manuscript.

### Conflict of interest statement

The authors declare that the research was conducted in the absence of any commercial or financial relationships that could be construed as a potential conflict of interest.
