# Supplementary material for: Culturally Appropriate Peer-Led Behavior Support Program for African Americans With Type 2 Diabetes
Source: Front Public Health. 2018 Nov 23;6:340. doi: 10.3389/fpubh.2018.00340 (PMC6265436; doi:10.3389/fpubh.2018.00340)
Supplement: Supplementary file 1 [file Table_1.DOCX]

**Supplement 1**

**Table 1**. Emotional support sub-themes with supporting quotes

| Sub-theme | Supporting Quotes |
| --- | --- |
| Listened, advised, and encouraged | a. “She’ll call and check up on me, listen to me and offer me some advice.”  b. “She always does listening to what you’ve got to say. And taking it into consideration and always giving a positive output.”  c. “I think when I first met Ms. …., I was going through some mental stuff and she talked to me, talked me down and got me started going to see somebody about the problems that I was having so it’s working out really well because I do feel better.” |
| Nonjudgmental | a. “Because you have some people that interview you and make you feel like you – big as I am – make you feel like you’re that small. She’s a good coach.”  b. “She offers me a lot of advice as far as when I first started with Ms. … I was sneaking eating and she would know it. One time she caught me. She didn’t get all aggressive or ugly like that. She just broke it down to me and brought it down to how important my health was and got me back on the right track.” |
| Provided information | a. “Because they’re pretty thorough in presenting to us the information.”  b. “My favorite support is her being there just to talk to me like if I run into a problem or if I don’t understand, I can call her and she’ll stop what she’s doing and just go over it over the phone with me and that’s my favorite support when she’s there for me.”  c. Emotional support is most important. “When we be in the class, peer supporters tell us different things then they go over it and manage it and then we talk. They explain different things about your vegetables and explain different types of things you go to do and what you’ve got to do and it really helps.” |
| Was there for me | a. “She has always given me her phone number. Like if I needed her – like falling down, or giving up – she was there to support me, to bring me back up. I've always looked at her in that way, of doing things where that I could count on her, and she would be there for me. Opened it up, that's right down.”  b. “Well, because she’s there for me and I like that because I can call her and say, “I’m having trouble like my glucose is not saying what it’s supposed to say.” And she would say, “Well check it again after you eat or check it before you eat this time.” “I can’t think of anything, she’s always there. At the end of the day I see some light at the end of that tunnel.”  c. “Well my favorite would be her calling me and telling me that we meeting this week. That would be my favorite because that I like. Like I said, I’m a go-getter and she’ll call me and let me know the next one. I like her really being there.”  d. “My favorite is ongoing support, to know that she’s going to be there still.” |
| Was a good supporter | a. “She always call us, and talk with us, and remind us when the class is and everything. She always a good supporter. She listen to everybody”  b. “Excellent, Ms. ….. Calls me all the time and sometimes she calls me twice a week just to check on me and see how I’m doing and if I need anything or if there’s anything she can do for me. She really supports us.”  c. “Emotional and clinical and just being supportive. Her calling me and sometimes I sit at home because I am disabled I be there by myself and I just be needing somebody to call and it seems like she knows and its Ms. … and it really helps a lot. The classes, they help a lot.”  d. “Like I said, emotional because you never know how a person is feeling and sometimes just one word can brighten that person’s day. When she calls it’s good to hear her voice.”  e. “Emotional because it’s good to have someone to talk to when I’m home all the time by myself. When Ms. ... calls, it just brightens my day up. She’s just one of those people that when you hear their voice, “Hello Ms.…,” it just brightens up my day.” |
| Was a kind person | “She’s very, very kind-hearted. She understands because it was many times that I would get aggravated because I would want to go walk. I would want to go this and I would want to go do that but physically I couldn’t do it. And it started touching me mentally and she’d always step in and come in with a positive role and just tell me, you know, look ahead because things are gonna get better and you’re gonna be getting to do this and it fell in place because I lost the weight and still losing. I just found myself continuing to do more and more that I wasn’t able to do and I could see that smile on her face. I told you so.” |
